# Comparison of service tactic formation on players’ movements and point outcome between national and beginner level padel

**DOI:** 10.1371/journal.pone.0250225

**Published:** 2021-10-27

**Authors:** Jesús Ramón-Llin, José Guzmán, Rafael Martínez-Gallego, Goran Vučković, Diego Muñoz, Bernardino J. Sánchez-Alcaraz

**Affiliations:** 1 Department of Musical, Plastic and Corporal Expression, Faculty of Education, University of Valencia, Valencia, Spain; 2 Department of Physical Education and Sport, Faculty of Sport Sciences, University of Valencia, Valencia, Spain; 3 Faculty of Sport, University of Ljubljana, Ljubljana, Slovenia; 4 Department of Musical, Plastic and Corporal Expression, Faculty of Sport Sciences, University of Extremadura, Cáceres, Spain; 5 Department of Physical Activity and Sport, Faculty of Sport Sciences, University of Murcia, San Javier, Spain; Universita degli Studi di Verona, ITALY

## Abstract

The aim of this study was to analyze the influence of service tactic formation on players’ movements and point outcome at two different performance levels. The sample contained 2,148 points corresponding to 18 matches from two male padel indoor tournaments. Players were classified according to their game level: high-level (N = 36; age = 33.3 ± 6.9 years) and beginner (N = 36; age = 35.4 ± 6.8 years). Variables pertaining to service tactic formation (conventional or Australian), point outcome and movement patterns were analysed from the matches through systematic observation. The results showed how high-level players used a significantly higher percentage of the Australian formation than beginners. Also, high-level players won a significantly higher percentage of points than recreational players when using both service tactics. According to movement variables, servers were significantly closer to the net and the side wall using a conventional formation when the returner hit the ball. Furthermore, servers had to move quicker when they used the Australian formation. Finally, the comparison of movement patterns of servers according to game level, showed how high-level players ran faster to the offensive position, covered a greater distance and spent less time between serve and return impacts than beginners.

## Introduction

Padel was born in Mexico approximately 50 years ago [1]. This sport is played in pairs (2 vs. 2), on a small-sized completely closed grass court (20 x 10 m), surrounded by glass and metallic mesh areas which the ball can bounce on [2]. In the last decade, there has been an enormous growth in the number of players, as it is practised in several countries around the world [3]. Research in padel has been mainly focused on describing match activity and detecting effective performance indicators [4–6] in three fundamental aspects: temporal structure [7–10], players’ movements and distance covered on the court [11–14] and game actions, such as technical or tactical parameters [5,6]. The results of these studies have shown that these variables may also differ depending on the gender or players’ levels [5,6].

However, no investigations have been found examining players’ serve statistics [15]. One of the most important performance indicators in racket sports, like tennis, is the serve [16]. In tennis, the serve lets the server win the point indirectly due to the advantage of the opponent’s imbalance after a great serve or directly through an ace [17]. Thus, tennis players win about 68% of points with the first serve, revealing differences between genders. In this case, the percentage is higher in men’s singles than women’s [16,18,19]. Previous studies found that the serve was more determinant in tennis doubles (played in pairs), likely due to the presence of the server’s partner covering the net [20]. In padel, one investigation showed that the serving pair could have a significant advantage in rallies, which lasted until shot 12 in men and shot 7 in women [21]. These results suggest that, in padel, the influence of the service could be related to the rally because the predominance of the “serve and volley” strategy allows the serving pair to adopt an offensive position and move close to the net first, as we indicate previously [4,22]. In this way, we observed that winners scored about 34% more points from the net than losers [4].

**Fig 1:**
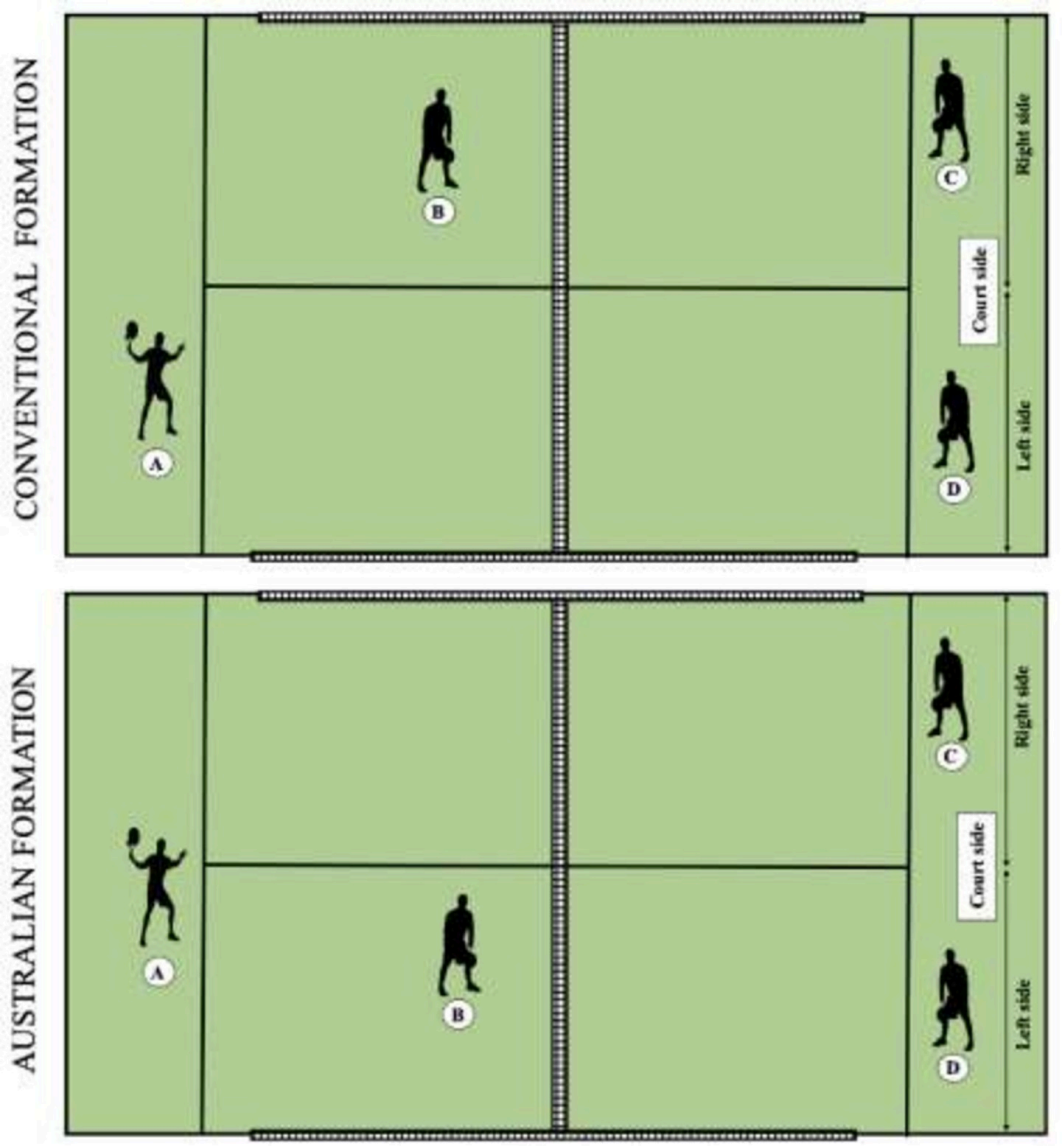
Service tactic formation. A = server; B = server’s partner; C = returner; D = returner’s partner.

Previous studies have analysed the movement patterns during the serve in racket sports [23,24], and have been discussed when comparing winning and losing players [25,26]. In squash, Vučković et al., [25] reported than winners covered more distance than losers because of the extra number of movements to the central area following a serve made by the winners, which was a consequence of them serving more often than the losers. Also, movement patterns may change according to players’ performance levels in padel. Another study reported that the server covers more distance than his partner at different performance levels, although percentages indicate a tendency to run more at the highest levels [22]. However, the serve in padel may be different from other racket sports, because of the rules of the game [2]. In padel, the serve must be an underhand shot from a bouncing ball hit from below waist level, so the ball cannot be hit as hard as in tennis. In addition, the serve-return shot is affected by the effect of the serve and the side wall, as we informed previously [15]. Also, the service pair can use two different service tactic formations (Fig 1): conventional (the server’s partner stands close to the net on the other side of the court to the server) or Australian (the server’s partner stands close to the net on the same side of the court as the server), irrespective of the side where the serve takes place [15]. A better knowledge of player’s behaviour (especially internal and external loading) when serving is extremely useful for designing specific-training exercises and developing appropriate game strategies [27,28]. However, there is very limited information about tactical and kinematic patterns during a padel match regarding service tactic formation. To this purpose, we aimed to analyse the influence of service tactic formation on players’ movements and point outcome between high level and beginner level padel. Such knowledge may have implications for designing accurate training drills close to real competitive situations.

## Material and methods

### Participants

The sample comprised 2,148 points from 18 matches of two male padel indoor tournaments, authorised by the Valencian Federation. Tournaments had different game categories: first category (high level players) and third and fourth category (beginner players). The matches were selected for convenience, involving quarter-final, semi-final or final rounds, to guarantee a greater equality for each group, due to last rounds are more intense than qualy rounds [29]. The main sample was classified according to players’ levels into two different categories: high-level (N = 36; Age = 33.3 ± 6.9 years; Hand dominance = 26 right-handed, 8 left-handed) and beginner (N = 36; Age = 35.4 ± 6.8 years; Hand dominance = 32 right-handed, 4 left-handed). High-level players had federal licences, and in the previous season had competed in first category in at least 8 national tournaments. Beginner players had at least two years of experience and practices padel for an average time of three hours per week. The inclusion criteria were: subjects have not recent injuries and they use both service tactics formation. Pairs using only one tactic formation did not were included. The written consent of the tournament organisers was obtained to film and analyse the matches. The study was approved by the University of Extremadura Research Ethics Committee (ref number: 154/2020) and all participants provided their written informed consent to participate in the current investigation.

### Variables

Variables can be classified as dependent or independent variables. The independent variables were service tactic formation and game level:

- Service tactic formation: Following Ramón-Llin et al. [15], players’ tactic was classified in two categories, Conventional formation (the server’s partner stands close to the net on the other side of the court to the server) or the Australian formation (the server’s partner stands close to the net on the same side of the court to the server), irrespective of the side where the serve takes place (Fig 1).
- Game level: Players were classified in two levels, high-level and beginners.

**Fig 2:**
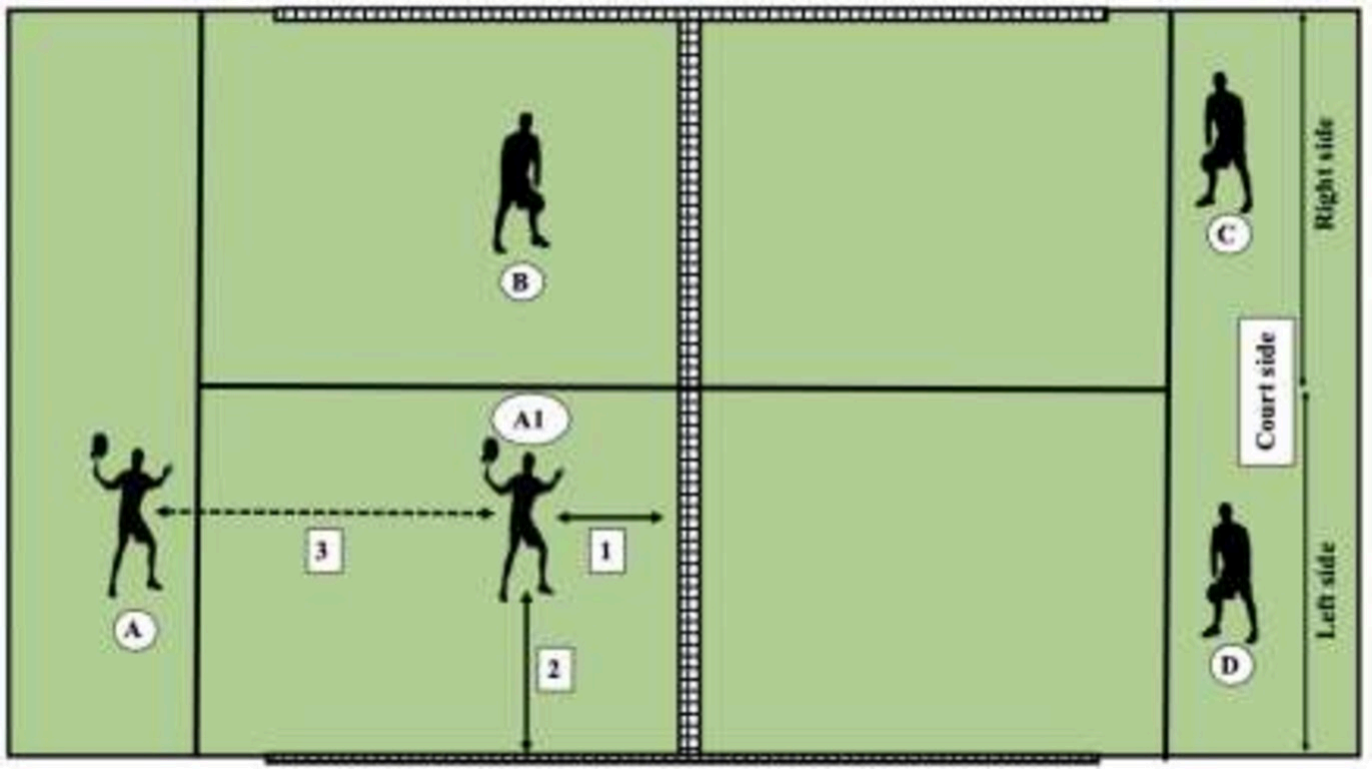
Movement variables. A = server; B = server’s partner; C = returner; D = returner’s partner; A1 = Server’s position when returner hits the ball; 1 = Server’s distance to the net when returner hits the ball; 2 = Server’s distance to the side wall when returner hits the ball; 3 = Server’s distance covered between serve and return.

Dependent variables were point outcome and movement variables:

- Point outcome: points were classified according to the winning or losing pair of the point (servers win and returning players win).
- Movement variables (Fig 2): The description of the variables was based on previous studies [14,15,22], distinguishing:
  ○ Distance covered by the server between serve and return stroke impacts (m).
  ○ The server’s distance to the net when the returner hits the ball (m).
  ○ The server’s distance to the closer side wall when the returner hits the ball (m).
  ○ The time between serve and return stroke impacts (s).
  ○ The server’s maximum velocity between serve and return stroke impacts (m/s).

### Procedure

Two digital Bosch Dinion Model IP 455 video cameras (Bosch, Munich, Germany) were used to film the matches (25 frames per second), sagittally placed over the courts at 6 m from the centre and over the service line. Players and ball coordinates were analysed using a computerized motion tracking system (SAGIT/Squash) [15,22], that uses computer vision methods on video captured via fixed cameras located above the court (Fig 1). The SAGIT/Squash tracking system has been specifically designed for racket sports analysis. In addition, the software allows the use of position inputs to track the ball location. A separate input system was designed to allow the operator watching the video from the overhead camera while highlighting the ball position on the court via a touch sensitive interface. The techniques for transferring video images into Tracker has been well documented [30]. Similarly, the reliability for the resultant calculations of players and ball position on court has been shown to be acceptable for analysis purposes [31]. Video analysis of service tactic formation, point outcome and players’ movements was conducted by systematic observation using the LINCE software [32]. This free-access software allows the creation of a coding tool synchronized with the video, and the resulting data file can be exported in Excel. Two observers specialised in padel (over 5 years’ experience) were specifically trained to perform the recording. The training focused on the clear identification of the variables and the use of the software. At the end of the training process, each observer analysed the same random sample of points (n = 250) in order to calculate the inter-observer reliability by means of Kappa Cohen, obtaining a very high agreement (k > 0.92) [33]. Finally, intra-observer evaluation was done at the end of the observation process by Cohen’s Kappa calculation, yielding a very good strength of agreement with scores over .98 [33].

### Data analysis

**Fig 3:**
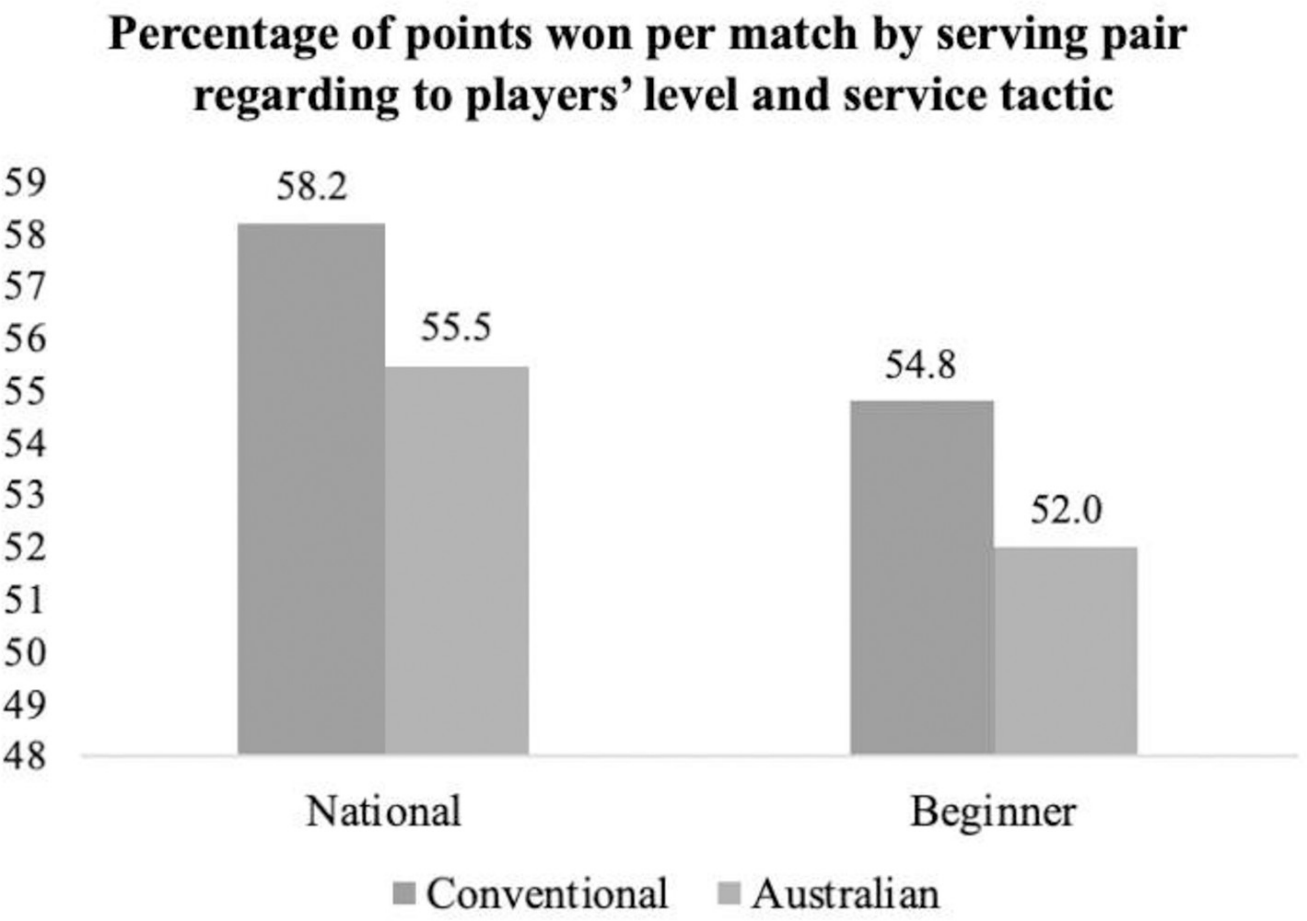
Percentage of points won per match by serving pair regarding players’ levels and service tactic formation.

The descriptive analysis included frequencies, means and standard deviations. Assumptions of normality and homogeneity of variances were verified using the Kolgomorov-Smirnov test and the Levene test. Symmetry was analysed dividing skewness and kurtosis by their respective standard error, getting distributions > 1.96. Then, non-parametric tests were implemented [34]. To analyse percentages for service tactic formation regarding to players’ levels (Table 1), a chi square analysis with the Bonferroni correction was performed to identify relationships, then post-hoc Z test were made to compare proportions of column between different game levels. To analyse differences in movement variables and compare them between serve tactic formation (Table 2) and game level (Table 3), Mann-Whitney tests were used with Bonferroni adjustment (p/number of comparatives). After Mann-Whitney tests, the effect size of these vaiables was calculated acording to Cohen´s d [35], (being <0.20, 0.20–0.50, 0.50–0.8 and >0.80 the thresholds for, trivial, small, moderate and high differences). Finally, it was analysed the differences of percentage of points won per match by the serving pair and compare them by game level and service tactic formation (Fig 3). Chi- square relationship tests with Bonferroni adjustment were also performed, and then Z-tests pos-hoc to compare column proportions. After chi- square and Z-tests, Cramer’s *V* was calculated to measure the strength of the relationship, considering small (*V* = .06), medium (*V* = .17) and large (*V* = .29) effects for df = 3 [36]. Significant values were considered for p < .05. Data were processed by IBM SPSS 20.0 Statistics for Macintosh (Armonk, NY: IBM Corp., USA).

**Table 1:** Percentages for service tactic formation regarding to players’ levels.

Service tactic formation	Game level		*p*
	High level N (%)	Beginner N (%)	
Conventional	575 (53.1)**a**	890 (83.5)**b**	.001**
Australian	507 (46.9)**a**	176 (16.5)**b**	

Note: N = Number; % = Percentage

** = p < .01

**a, b** = indicate significant differences in the Z tests for comparison of column proportions from p < .05 adjusted according to Bonferroni.

**Table 2:** Differences in movement variables between Australian and conventional serve tactics regarding players’ levels.

Players’ levels	Movement variables	Serve tactic formation						*p*	*d*
		Australian			Conventional				
		M	Mn	SD	M	Mn	SD		
High level	Server’s distance to the net (m)	5.12	5.17	.60	5.04	5.04	.55		
	Time between serve and return stroke impacts (s)	1.22	1.2	.19	1.22	1.2	.20		
	Server’s distance to the side wall (m)	3.60	3.7	.60	3.45	3.58	.61	**	.25
	Server’s maximum velocity (m/s)	3.21	3.23	.34	2.69	2.69	.35	**	1.51
	Distance covered by server (m)	2.97	2.93	.54	2.48	2.44	.51	**	.93
Beginner	Server’s distance to the net (m)	6.11	6.03	1.29	6.54	6,75	1.27	**	.34
	Time between serve and return stroke impacts (s)	1.45	1.4	.26	1.42	1.4	.24		
	Server’s distance to the side wall (m)	3.72	3.72	.46	3.63	3.68	.53		
	Server’s maximum velocity (m/s)	2.43	2.48	.81	1.74	1.52	.75	**	.88
	Distance covered by server (m)	2.49	2.44	1.11	1.69	1.47	.92	**	.78

Note

* *p* < .01

** *p* < .001; d = Effect size.

**Table 3:** Differences in movement variables regarding players’ levels.

Movement variables	Players’ level							
	High level			Beginner				
	M	Mn	SD	M	Mn	SD	*p*	*d*
Server’s distance to the net (m)	5.08	5.07	.58	6.47	6.7	1.28	**	1.40
Time between serve and return stroke impacts (s)	1.22	1.20	.20	1.42	1.4	.25	**	.88
Server’s distance to the side wall (m)	3.52	3.64	.61	3.64	3.69	.52	**	.21
Server’s maximum velocity (m/s)	2.93	2.93	.43	1.85	1.7	.80	**	1.68
Distance covered by server (m)	2.71	2.64	.58	1.82	1.62	1.00	**	1.09

Note

* *p* < .01

** *p* < .001. d = Effect size.

## Results

Table 1 shows the differences in service tactic formation in relation to players’ levels (*χ^2^* = 322.9; *p <* .001; *V* = .31). High-level players used a significatively higher percentage of the Australian formation than beginners. At the high level, players used both service tactic formation interchangeably, but at lower levels, players preferred to use the conventional formation when serving.

Fig 3 shows the relationship between the percentage of points won per match by the serving pair, and the level of the players, in relation to service tactic formation (*χ*^2^(3) = 2.34; *p* = .51). Results showed non-significant relationship, although high level players won a higher percentage of points than recreational players when using both service tactics: Australian (*χ*^2^(1) = .53; *p* = .47) and conventional (*χ*^2^(1) = 1.31; *p* = .25). In addition, the serving pair won more points when they used the conventional formation at both levels, but no significant differences were found at the high level (*χ*^2^(1) = .797; *p* = .372) or beginner level (*χ*^2^(1) = .586; *p* = .444).

Table 2 shows differences in movement variables between Australian and conventional serve tactics regarding players’ levels. The results showed how the server was significantly closer to the net using a conventional formation when the returner hits the ball at the beginner level (U = 63543; Z = -4.0; *p* < .001; *d* = .34) and almost significantly at the high level (U = 1333599; Z = -2.3; *p* = .02). Also, the server was significantly closer to the side wall using a conventional formation when the returner hits the ball at the high level (U = 124651; Z = -4.07; *p* < .001; *d* = .25). However, servers were farther away from offensive positions using an Australian formation, and they had to run faster to the net after hitting the ball at the high level (U = 42304; Z = -20.2; *p* < .001; *d* = 1.51) and beginner level (U = 41720; Z = -9.8; *p* < .001; *d* = .88). Finally, servers covered significantly more distance after serving in the Australian formation at the high level (U = 71734; Z = -14.4; *p* < .001; *d* = .93) and beginner level (U = 45787; Z = -8.7; *p* < .001; *d* = .78).

Table 3 shows differences in movement variables between high level and beginner players. High level servers are closer to the net (U = 221718; Z = -24.7; P < 0.001; *d* = 1.40) and the side wall (U = 523924; Z = -3.6; *p* < .001; *d* = .21) when the returner hits the ball, run faster to the offensive position (U = 160369; Z = -29.0; *p* < .001; *d* = 1.68), cover a greater distance (U = 262245; Z = -21.8; *p* < .001; *d* = 1.09) and spend less time between serve and return impacts (U = 253964; Z = -22.5; *p* < .001; *d* = .88) than beginner players.

## Discussion

The aim of this study was to analyse the influence of service tactic formation on players’ movements and point outcome at two different performance levels. Results provide a novel insight into the serve statistics in padel players. Current findings showed significant differences in service tactic formation according to players’ levels. Beginners used the conventional formation in more than 80% of the points, while high-level padel players used both service tactic formations interchangeably. Usually, high level players alternate the use of the Australian and conventional formation at the beginning of the points, allowing to start the point on the same side of the net. This suggests that, in elite padel, each player is specialised in one side of the court [37,38], coinciding with other sports, such as soccer [39], basketball [40] or rugby [41], which reveal tactical specialisation depending on the playing position. However, the results obtained in this study highlighted the importance that padel players be able to play both side of the court, since more points are obtained with the conventional formation.

Regarding point outcome, serving pair won more points than returners in both competitive levels. The service has been shown to be advantageous in padel [15,21] and similar racket sport such us doubles tennis [42,43]. Serve tends to increase the probability of winning the rally, but the extent of this advantage seems to be determined by players’ level or service tactic formation. Current results showed that high-level padel players won a higher percentage of points than beginners when using both service tactics: Australian and conventional, but without significant differences. More than 70% of the rallies in padel are resolved within the first 9 seconds [7,32], and the serve allows players to start the point in the net area, where the vast majority of points are scored (80%) [4]. This higher percentage of points won by high-level players is to be expected due to greater technical superiority when they hit volleys or smashes [43–46]. Also, servers won between 2–3% more points using the conventional formation. This could be related with movement variables [47]. Results showed how servers were significantly closer to the net and the side wall using a conventional formation when the returner hits the ball, as the server must cover more distance using the Australian formation. Net distance when the player hits the ball is an important predictor of shot outcome. In the same way like tennis [48,49] or badminton [29], some previous studies highlighted that the highest percentage of winners appears in areas close to the net, at a distance of between 2 and 4 meters from the net [50,51]. Furthermore, servers had to move quicker when they used the Australian formation. A higher maximum speed when players move to the ball will also require greater braking, which may affect the player’s balance at the moment of hitting [52,53] and increase the probability of making a mistake [54]. These findings suggest that coaches should consider training conditioning sessions [55–57] focusing on movements when players serve (specific distance, maximum speed, acceleration and deceleration). Interestingly, if we compare these results to previous studies in doubles tennis, we see that conventional formation prevails in professional tennis, however, Australian and I-formation had higher efficiency, especially in male category [57] especially when serving next to the lead. Specifically, Kocib et al. [58] pointed out that the men did not use the Australian tactic, and although the I-formation was more effective than the Classical one, a slightly greater use of the conventional tactic prevailed. Therefore, it seems that, as in high-level padel the conventional tactic is combined with the Australian, without using the I-formation, in tennis the classic tactic (equivalent to the conventional in padel) is combined with the I-formation, without using the australian. These differences can be explained due to the characteristics of tennis, as the higher serve velocity and the inexistence walls to bounce the ball after the serve, so serve in padel is less offensive [15]. Also, this serve efficiency in tennis vary depending on gender, serving side or game score [58,59], so future research in padel is needed to well define effectiveness of service tactic formations.

Finally, the comparison of movement patterns of servers according to game level showed how high-level players ran faster to the offensive position, covered a greater distance and spent less time between serve and return impacts than beginners. These differences suggest that, at a higher level of performance, players have a better physical condition, are faster and cover greater distances, as indicated by some studies in tennis [60] or squash [30]. Also, in padel, a previous study highlighted a higher game pace and maximum speed when the player’s game level increases [61]. However, it seems that during the full point, high-level padel players cover less distance and at a lower speed compared with amateur players [62], indicating better on-court positioning with respect to the ball. This leads to the suggestion that more specific factors such as acceleration, agility, balance and response time should merit special attention in physical padel training, as we reported [63,64].

The current study adds novel insights into the growing body of knowledge of in padel training and assessment, providing for the first time an analysis of service tactic formation and its relationship with players’ movements and positions in two different game levels. Such knowledge may constitute a very important factor affecting tactics training and conditioning in padel players. The findings of this study suggest, as a practical application, that coaches should consider using game-based approach exercises, using different serving tactic formation, so this would help players for better making decisions and movements according to opponents or partner positioning. However, some limitations to the study should be noted. First, contextual variables such as match status were not recorded. Given the influence of situational variables on game performance [65,66], it would be very interesting to include such information in future research on padel. Moreover, other variables that may affect serve statistics, such as serve speed, spin or directions and players’ hand dominance [67–69] have not been taken into account. Finally, the number of matches analysed could not be representative of the two-level performance, so futures studies must increase the sample size.

## Conclusions

In this work we aimed to advance the emerging field of analysis of padel on a collective scale. This study reports novel contributions on spatial positionings and movements indicators in padel. Our data suggest that service tactic formation had a significant influence on players’ movements and could affect point outcome between high-level and beginner level padel. Specifically, high-level padel players uses indistinctly both tactics formations, while beginner players used a significantly higher percentage of conventional formation. Conventional formation allowed severs to be closer to the net in high level, and closer to the side wall (when returner hits the ball) in both levels. Furthermore, servers had to move quicker when they used the Australian formation. Finally, the comparison of movement patterns of servers according to game level showed how high-level players run faster to the offensive position, covered a greater distance and spent less time between serve and return impacts than beginners.

## Supporting information

S1 File (SAV)

## References

[1] Sánchez-Alcaraz BJ. History of padel. Mater para la Hist del Deport. 2013;11:57–60.

[2] International Padel Federation. Rules of padel. FIP, redaktors. Lausanne; 2020.

[3] Courel-Ibáñez J, Sánchez-Alcaraz BJ, García S, Echegaray M. Evolution of padel in spain according to practitioners’ gender and age [Evolución del pádel en España en función del género y edad de los practicantes]. Cult Cienc y Deport. 2017;12(34):39–46.

[4] Courel-Ibáñez J, Sánchez-Alcaraz JB, Cañas J. Effectiveness at the net as a predictor of final match outcome in professional padel players. Int J Perform Anal Sport. 2015;15(2):632–40.

[5] Torres-Luque G, Ramirez A, Cabello-Manrique D, Nikolaidis TP, Alvero-Cruz JR. Match analysis of elite players during paddle tennis competition. Int J Perform Anal Sport. 2015;15(3):1135–44.

[6] Courel-Ibáñez J, Sánchez-Alcaraz BJ, Muñoz D. Exploring game dynamics in padel: implications for assessment and training. J Strength Cond Res. 2019;33(7):1971–7. doi: 10.1519/JSC.0000000000002126 28723819

[7] Courel-Ibáñez J, Sánchez-Alcaraz BJ. Effect of situational variables on points in elite padel players [Efecto de las variables situacionales sobre los puntos en jugadores de pádel de élite]. Apunt Educ Física y Deport. 2017;127(1):68–74.

[8] Courel-Ibáñez J, Sánchez-Alcaraz BJ, Cañas J. Game Performance and Length of Rally in Professional Padel Players. J Hum Kinet. 2017;55:161–9. doi: 10.1515/hukin-2016-0045 28210348 PMC5304268

[9] Pradas F, Cachón J, Otín D, Quintas A, Arraco SI, Castellar C. Anthropometric, physiological and temporal analysis in elite female paddle players. Retos, Nuevas Tendencias en Deport Educ Física y Recreación. 2014;25:107–22.

[10] Muñoz D, García A, Grijota FJ, Díaz J, Sánchez IB, Muñoz J. Influence of set duration on time variables in paddle tennis matches [Influencia de la duración del set sobre variables temporales de juego en pádel]. Apunt Educ Fis y Deport. 2016;123:69–75.

[11] Priego JI, Olaso J, Llana S, Pérez P, Gonález JC, Sanchís M. Padel: a quantitative study of the shots and movements in the high-performance. J Hum Sport Exerc. 2013;8(4):925–31.

[12] Ramón-Llin J, Guzmán JF, Martinez-Gallego R, Vučković G, James N. Time-motion analysis of Pádel players in two matches of the 2011 pro tour. No: Peter D., O’Donoghue PG, redaktors. Performance Analysis of Sport IX. London: Routledge; 2014.

[13] Amieba C, Salinero Martín J. General aspects of paddle tennis competition and its physiological demands [Aspectos generales de la competición del pádel y sus demandas fisiológicas]. Agon Int J Sport Sci. 2013;3(2):60–7.

[14] Ramón-Llin J, Guzmán J, Llana S, Vuckovic G, Muñoz D, Sánchez-Alcaraz BJ. Analysis of distance covered in padel based on level of play and number of points per match [Análisis de la distancia recorrida en pádel en función del nivel de juego y el número de puntos por partido]. Retos Nuevas Tendencias en Educ Fis Deport y Recreacion. 2020;39:205–9.

[15] Ramón-Llin J, Guzmán JF, Llana S, Martínez-Gallego R, James N, Vučković G. The Effect of the Return of Serve on the Server Pair’s Movement Parameters and Rally Outcome in Padel Using Cluster Analysis. Front Psychol. 2019;10:1–8. doi: 10.3389/fpsyg.2019.00001 31191397 PMC6546820

[16] Gillet E, Leroy D, Thouvarecq R, Stein JF. A notational analysis of elite tennis serve and serve-return strategies on slow surface. J Strength Cond Res. 2009;23(2):532–9. doi: 10.1519/JSC.0b013e31818efe29 19197212

[17] Reid M, McMurtrie D, Crespo M. The relationship between match statistics and top 100 ranking in professional men’s tennis. Int J Perform Anal Sport. 2010;10(2):131–8.

[18] O’Donoghue P. The most important points in grand slam singles tennis. Res Q Exerc Sport. 2001;72(2):125–31. doi: 10.1080/02701367.2001.10608942 11393875

[19] Hizan H, Whipp P, Reid M. Comparison of serve and serve return statistics of high performance male and female tennis players from different age-groups. Int J Perform Anal Sport. 2011;11(2):365–75.

[20] Furlong J. The service in lawn tennis: how important is it? No: Reilly T, Hughes MD, Lees A, redaktors. Science and Racket Sports I. London: E &FN Spon; 1995.

[21] Sánchez-Alcaraz BJ, Muñoz D, Pradas F, Ramón-Llin J, Cañas J, Sánchez-Pay A. Analysis of serve and serve‐return strategies in elite male and female padel. Appl Sci. 2020;10(19):6693.

[22] Ramón-Llin J, Guzmán J, Llana S, Vučković G, James N. Comparison of distance covered in paddle in the serve team according to performance level. J Hum Sport Exerc. 2013;8(Proc3):S738–42.

[23] Hughes M, Moore P. Movement analysis of elite level male “serve and volley” tennis players. No: Hughes M, Maynard I, Lees A, Reilly T, redaktors. Science and Racket Sports II. London: Routledge; 1998.

[24] Kilit B, Arslan E, Soylu Y. Time-motion characteristics, notational analysis and phyisiological demands of tennis match play: a review. Acta Kinesiol. 2018;12(2):5–12.

[25] Vuckovic G, Dezman B, Erculj F, Kovacic S, Pers J. Differences between the winning and the losing players in a squash game in terms of distance covered. No: Lees A, Khan JF, Maynard IW, redaktors. Science and Racket Sports III. London: Routledge; 2004.

[26] Kilit B, Arslan E. Physiological responses and time-motion characteristics of young tennis players: comparison of serve vs. return games and winners vs. losers matches. Int J Perform Anal Sport. 2017;17(5):684–94.

[27] Butterworth A, O’Donoghue P, Cropley B. Performance profiling in sports coaching: a review. Int J Perform Anal Sport. 2013;13(3):572–93.

[28] Kilit B, Şenel Ö, Arslan E, Can S. Physiological responses and match characteristics in professional tennis players during a one-hour simulated tennis match. J Hum Kinet. 2016;50(2):83–92. doi: 10.1515/hukin-2015-0173 28149371 PMC5260553

[29] Chiminazzo JGC, Barreira J, Luz LSM, Saraiva WC, Cayres JT. Technical and timing characteristics of badminton men’s single: comparison between groups and play-offs stages in 2016 Rio Olympic Games. Int J Perform Anal Sport. 2018;18(2):245–54.

[30] Vučković G, Perš J, James N, Hughes M. Tactical use of the T area in Squash by players of differing standard. J Sport Sci. 2009;27:863–71. 10.1080/02640410902926412 19551552

[31] Vučković G, Perš J, James N, Hughes M. Measurement error associated with the Sagit/squash computer tracking software. Eur J Sport Sci. 2010;10:129–40.

[32] Gabin B, Camerino O, Anguera MT, Castañer M. Lince: Multiplatform Sport Analysis Software. Procedia - Soc Behav Sci. 2012;46:4692–4.

[33] Altman DG. Practical statistics for medical research. London: Chapman and Hall; 1991.

[34] Pallant J. SPSS Survival Manual: A step by step guide to data analysis using SPSS program. Australia: Alen & Unwin; 2011.

[35] Cohen J. Statistical power analysis for the behavioral sciences. 2nd red. Hillsdale NJ: Erlbaum; 1988.

[36] Fritz CO, Morris PE, Richler JJ. Effect size estimates: current use, calculations, and interpretation. J Exp Psychol Gen. 2012;141(1):2–18. doi: 10.1037/a0024338 21823805

[37] Ramón-Llin J, Guzmán J, Martínez-Gallego R, Muñoz D, Sánchez-Pay A, Sánchez-Alcaraz BJ. Stroke analysis in padel according to match outcome and game side on court. Int J Environ Res Public Health. 2020;17(21):7838. doi: 10.3390/ijerph17217838 33114684 PMC7662292

[38] Ramón-Llin J, Guzmán J, Martínez-Gallego R, Muñoz D, Sánchez-Pay A, Javier Sánchez-Alcaraz B. Análisis de la situación en la pista de los jugadores en el saque y su relación con la dirección, el lado de la pista y el resultado del punto en pádel de alto nivel (Analysis of the situation on the court of the players in the serve and its relationship. Retos. 2021;41:399–405.

[39] Andrzejewski M, Chmura J, Pluta B, Strzelczyk R, Andrzej K. Analysis of sprinting activities of professional soccer players. J Strength Cond Res. 2013;27(8):2134–40. doi: 10.1519/JSC.0b013e318279423e 23168374

[40] Torres-Ronda L, Ric A, Llabres-Torres I, De Las Heras B, Schelling I Del Alcazar X. Position-Dependent Cardiovascular Response and Time-Motion Analysis During Training Drills and Friendly Matches in Elite Male Basketball Players. J Strength Cond Res. 2016;30(1):60–70. doi: 10.1519/JSC.0000000000001043 26284807

[41] Austin DJ, Kelly SJ. Positional differences in professional rugby league match play through the use of global positioning systems. J Strength Cond Res. 2013;27(1):14–9. doi: 10.1519/JSC.0b013e31824e108c 22344046

[42] Martínez-Gallego R, Crespo M, Jiménez J. Analysis of the differences in serve effectiveness between Billie Jean King Cup (former Fed Cup) and Davis Cup doubles tennis matches. Int J Sport Sci Coach. 2021; 16(3):777–83.

[43] Martínez-Gallego R, Crespo M, Ramón-Llin J, Micó S, Guzmán JF. Men’s doubles professional tennis on hard courts: Game structure and point ending characteristics. J Hum Sport Exerc. 2020;15(3):633–42.

[44] Courel-Ibáñez J, Sánchez-Alcaraz Martínez BJ, Cañas J. Game performance and length of rally in professional padel players. J Hum Kinet. 2017;55(1). doi: 10.1515/hukin-2016-0045 28210348 PMC5304268

[45] Sánchez-Alcaraz BJ, Courel-Ibañez J, Cañas J. Valoración de la precisión de golpeo en jugadores de pádel en función de su nivel de juego [Groundstroke accuracy assesment in padel players according to their level of play]. RICYDE Rev Int Ciencias del Deport. 2016;XII(45):324–33.

[46] Rivilla-García J, Moreno AM, Lorenzo J, Van den Tillaar R, Navandar A. Influence of opposition on overhead smash velocity in padel players. Kinesiology. 2019;51(2):206–12.

[47] Sánchez-Alcaraz BJ, Martínez-Gallego R, Llana S, Vučković G, Muñoz D, Courel-Ibáñez J, u.c. Ball Impact Position in Recreational Male Padel Players: Implications for Training and Injury Management. Int J Environ Res Public Health. 2021;18(2):435. 10.3390/ijerph18020435 PMC7828082 33430496

[48] Martínez-Gallego R, Guzmán JF, Crespo M, Ramón-Llin J, Vučković G. Technical, tactical and movement analysis of men’s professional tennis on hard courts. J Sports Med Phys Fitness. 2019;50–6. doi: 10.23736/S0022-4707.17.07916-6 29111626

[49] Martinez-Gallego R, Guzmán JF, James N, Ramón-Llín J, Crespo M, Vuckovic G. The relationship between the incidence of winners/errors and the time spent in different areas of the court in elite tennis. J Hum Sport Exerc. 2013;8(3):S601–7.

[50] Sánchez-Alcaraz BJ, Jiménez V, Muñoz D, Ramón-Llin J. Effectiveness and distribution of attack strokes to finsih the point in professional padel. Rev Int Med y Ciencias la Act Fis y del Deport; in press.

[51] Sánchez-Alcaraz BJ, Perez-Puche DT, Pradas F, Ramón-Llin J, Sánchez-Pay A, Muñoz D. Analysis of Performance Parameters of the Smash in Male and Female Professional Padel. Int J Enviromental Res Public Heal. 2020;17(7027):1–10. doi: 10.3390/ijerph17197027 32992940 PMC7579567

[52] Dos Santos T, Thomas C, Jones PA, Comfort P. Mechanical Determinants of Faster Change of Direction Speed Performance in Male Athletes. J Strength Cond Res. 2017;31(3):696–705. doi: 10.1519/JSC.0000000000001535 27379954

[53] Dos Santos T, Thomas C, Comfort P, Jones PA. The Effect of Angle and Velocity on Change of Direction Biomechanics: An Angle-Velocity Trade-Off. Sport Med. 2018;48(10):2235–53. 10.1007/s40279-018-0968-3 PMC6132493 30094799

[54] Martínez-Gallego R, Guzmán JF, James N, Pers J, Ramón-Llín J, Vučković G. Movement Characteristics of Elite Tennis Players on Hard Courts with Respect to the Direction of Ground Strokes. J Sports Sci Med. 2013;12(2):275–81. 24149806 PMC3761832

[55] Castellar C, Pradas F, Quintas A, Arraco S, Pérez JB. Perfil condicional de jugadoras de pádel de élite. Rev Andaluza Med del Deport. 2015;8(4):185.

[56] Carbonel JA, Ferrandiz J, Pascual N. Análisis de la frecuencia cardíaca en el pádel femenino amateur. Retos Nuevas Tendencias en Educ Fis Deport y Recreacion. 2017;(32):204–7.

[57] Llin Mas JR, Guzmán Luján JF, Martínez Gallego R. Comparación de la frecuencia cardiaca en competición, entre jugadores de pádel de elite y de categoría nacional. Retos Nuevas Tendencias en Educ Fis Deport y Recreacion. 2018;(33):91–5.

[58] Carboch J, Kocib T, Pechacova. Analysis of tactical variants in men’s and women’s tennis doubles on the international level. Asian J Sci Technol. 2014;5(3):204–7.

[59] Kocib T, Carboch J, Cabela M, Kresta J. Tactics in tennis doubles: analysis of the formations used by the serving and receiving teams. Int J Phys Educ Fit Sport. 2020;9(2):45–50.

[60] Fernandez-Fernandez J, Sanz-Rivas D, Sanchez-Muñoz C, Pluim BM, Tiemessen I, Mendez-Villanueva A. A comparison of the activity profile and physiological demands between advanced and recreational veteran tennis players. J Strength Cond Res. 2009;23(2):604–10. doi: 10.1519/JSC.0b013e318194208a 19197208

[61] Ramón-Llín J, Guzmán JF, Llana S, James N, Vučković G. Analysis of padel rally caracteristics for three competitive levels. Kinesiol Slov. 2017;23(3):39–49.

[62] Castillo-Rodríguez A, Alvero-Cruz JR, Hernández-Mendo A, Fernández-García JC. Physical and physiological responses in paddle tennis competition. Int J Perform Anal Sport. 2014;14(2):524–34.

[63] Sánchez-Alcaraz BJ, Orozco V, Courel-Ibáñez J, Sánchez-Pay A. Speed, agility, and strength assessment in young padel players. Retos. 2018;34:263–6.

[64] Navas D, Veiga S, Navarro E, Ramón-Llin J. Differences in kinematic and match-play demands between elite winning and losing wheelchair padel players. PLoS One. 2020;15(9 September):1–8. doi: 10.1371/journal.pone.0233475 32946458 PMC7500611

[65] Gómez MÁ, Lago-Peñas C, Pollard G. Situational variables. No: McGarry T, O’Donoghue P, Sampaio J, redaktors. Routledge handbook of sports performance analysis. London: Routledge; 2003. lpp. 259–69.

[66] Escudero-Tena A, Fernández-Cortes J, García-Rubio J, Ibáñez SJ. Use and efficacy of the lob to achieve the offensive position in women´s professional padel. Analysis of the 2018 WPT finals. Int J Environ Res Public Health. 2020;17(11):4061. 10.3390/ijerph17114061 PMC7312421 32517261

[67] Courel-Ibáñez J, Sánchez-Alcaraz BJ. The role of hand dominance in padel: performance profiles of professional players. Motricidade. 2018;14(4):33–41.

[68] O’Donoghue P, Ballantyne A. The impact of speed of service in Grand Slam singles tennis. No: Lees A, Kahn J., Maynard I, redaktors. Science and Racket Sports III. London; 2008. lpp. 179–84.

[69] O’Donoghue P, Brown E. The importance of service in Grand Slam singles tennis. Int J Perform Anal Sport. 2008;8(3):70–8.

